# Supplementary figures and images for: A Comparative Study of Techniques for Differential Expression Analysis on RNA-Seq Data
Source: PLoS One. 2014 Aug 13;9(8):e103207. doi: 10.1371/journal.pone.0103207 (PMC4132098; doi:10.1371/journal.pone.0103207)

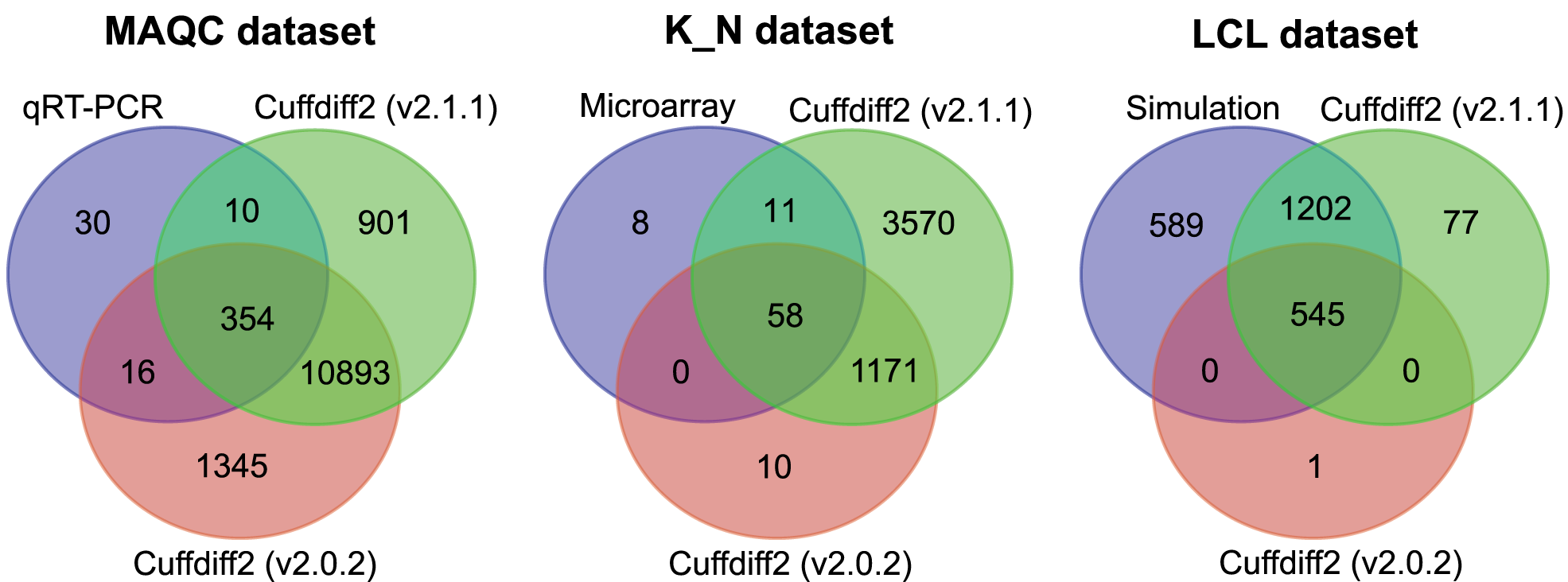

Supplement: Supporting Information S1 — Figure S1, Venn diagram showing the number of differentially expressed genes identified by two versions of Cuffdiff2. Figure S2, The effects of biological replicates on the differential expression analysis for Cuffdiff v2.0.2. Figure S3, The detected fold changes of all the differentially expressed genes identified by three tools were compared and shown, including DESeq vs. edgeR (top panel), DESeq vs. Cuffdiff2 (middle panel) and edgeR vs. Cuffdiff2 (bottom panel). File S1, Analysis pipelines, methods and examples of commands for differential expression analysis, subsampling fastq files and generating SAM/BAM files based on simulated count values. File S2, The raw count values for genes with high fold changes were picked up by edgeR but not by DESeq. Genes with high fold changes (the absolute value of log2 fold changes larger than 2) identified as DEGs by edgeR but not by DESeq are listed in the file. The gene ID, the log2 fold changes (logFC) and FDR from DESeq, the logFC and FDR from edgeR, the raw count values for the four replicates of sample K (K1–K4) and sample N (N1–N4) are shown in each of the columns. Table S1, Numbers of reads for the human hbr and uhr samples from the MAQC dataset. Table S2, Numbers of reads for the mouse neurosphere samples for treatment groups of K and N (the K_N dataset). Table S3, The number of reads for each individual sample of the LCL3 dataset. Table S4, The definition for TP, FP, TN, FN, TPR and FPR. Table S5, The false positive rate for Cuffdiff2, DESeq and edgeR based on the LCL1 dataset. (ZIP) [file pone.0103207.s001.zip › Supporting Information S1/Figure S1.tif]

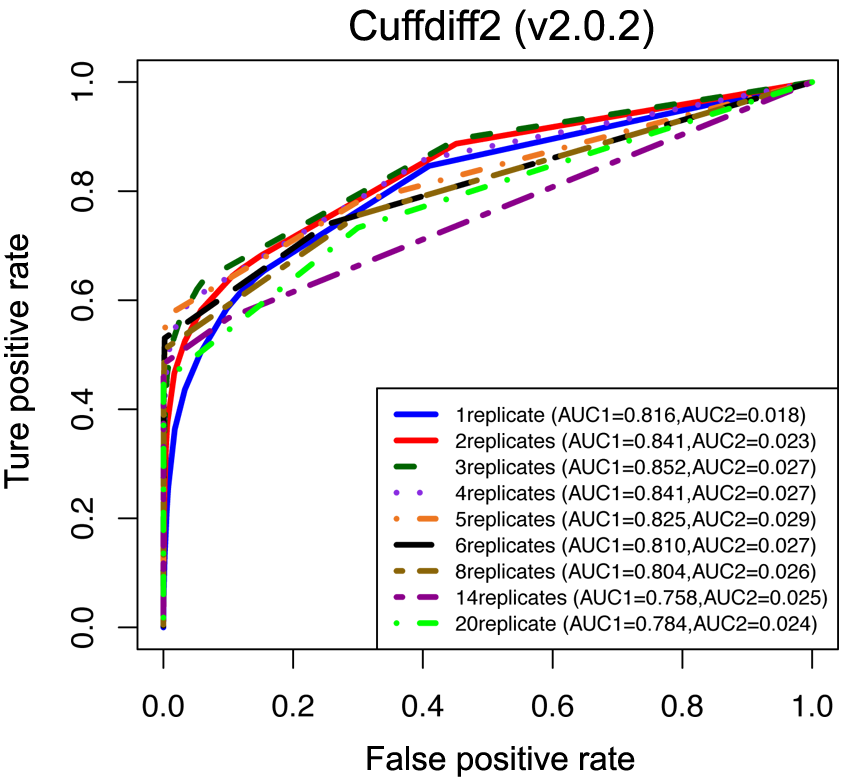

Supplement: Supporting Information S1 — Figure S1, Venn diagram showing the number of differentially expressed genes identified by two versions of Cuffdiff2. Figure S2, The effects of biological replicates on the differential expression analysis for Cuffdiff v2.0.2. Figure S3, The detected fold changes of all the differentially expressed genes identified by three tools were compared and shown, including DESeq vs. edgeR (top panel), DESeq vs. Cuffdiff2 (middle panel) and edgeR vs. Cuffdiff2 (bottom panel). File S1, Analysis pipelines, methods and examples of commands for differential expression analysis, subsampling fastq files and generating SAM/BAM files based on simulated count values. File S2, The raw count values for genes with high fold changes were picked up by edgeR but not by DESeq. Genes with high fold changes (the absolute value of log2 fold changes larger than 2) identified as DEGs by edgeR but not by DESeq are listed in the file. The gene ID, the log2 fold changes (logFC) and FDR from DESeq, the logFC and FDR from edgeR, the raw count values for the four replicates of sample K (K1–K4) and sample N (N1–N4) are shown in each of the columns. Table S1, Numbers of reads for the human hbr and uhr samples from the MAQC dataset. Table S2, Numbers of reads for the mouse neurosphere samples for treatment groups of K and N (the K_N dataset). Table S3, The number of reads for each individual sample of the LCL3 dataset. Table S4, The definition for TP, FP, TN, FN, TPR and FPR. Table S5, The false positive rate for Cuffdiff2, DESeq and edgeR based on the LCL1 dataset. (ZIP) [file pone.0103207.s001.zip › Supporting Information S1/Figure S2.tif]

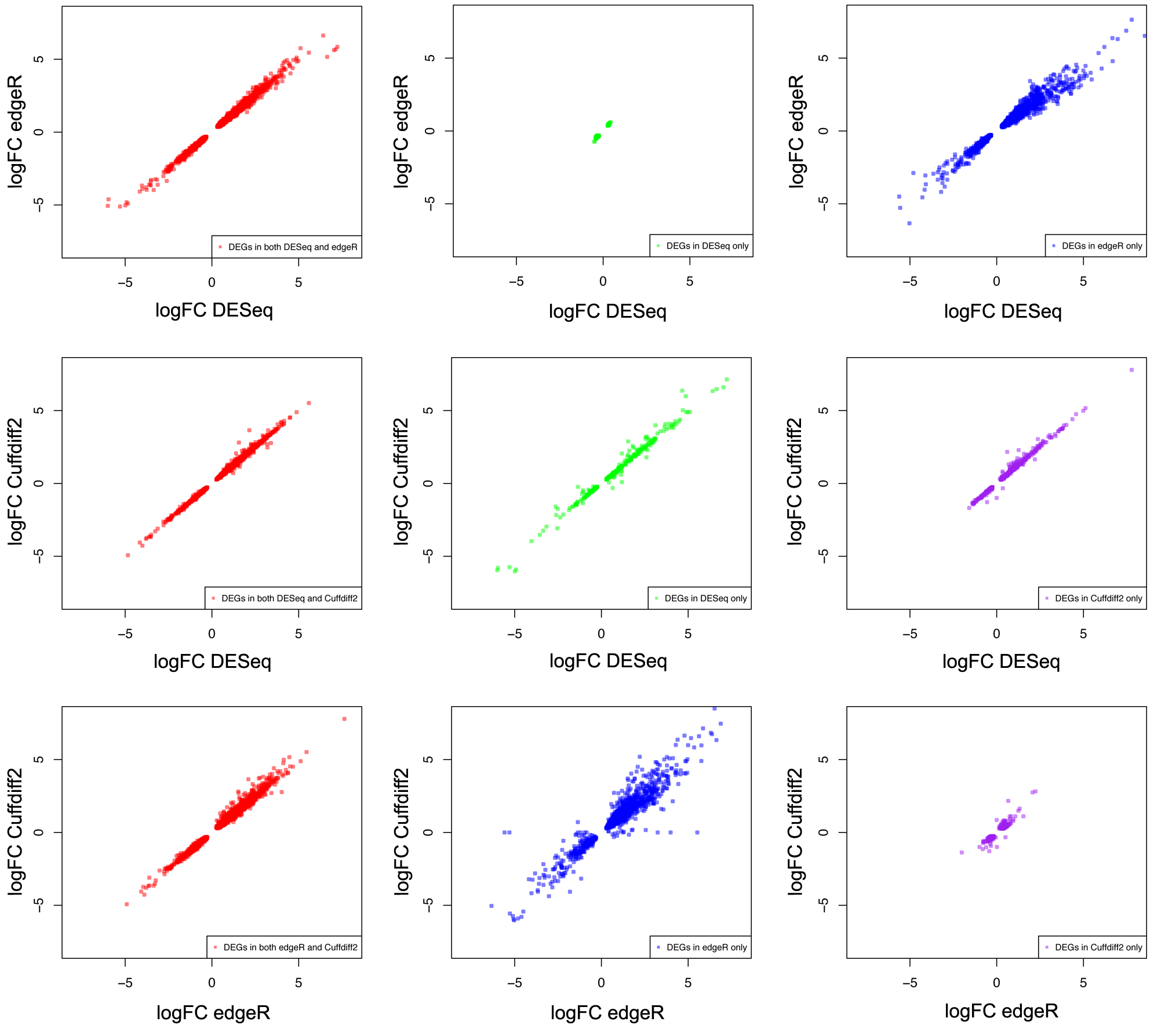

Supplement: Supporting Information S1 — Figure S1, Venn diagram showing the number of differentially expressed genes identified by two versions of Cuffdiff2. Figure S2, The effects of biological replicates on the differential expression analysis for Cuffdiff v2.0.2. Figure S3, The detected fold changes of all the differentially expressed genes identified by three tools were compared and shown, including DESeq vs. edgeR (top panel), DESeq vs. Cuffdiff2 (middle panel) and edgeR vs. Cuffdiff2 (bottom panel). File S1, Analysis pipelines, methods and examples of commands for differential expression analysis, subsampling fastq files and generating SAM/BAM files based on simulated count values. File S2, The raw count values for genes with high fold changes were picked up by edgeR but not by DESeq. Genes with high fold changes (the absolute value of log2 fold changes larger than 2) identified as DEGs by edgeR but not by DESeq are listed in the file. The gene ID, the log2 fold changes (logFC) and FDR from DESeq, the logFC and FDR from edgeR, the raw count values for the four replicates of sample K (K1–K4) and sample N (N1–N4) are shown in each of the columns. Table S1, Numbers of reads for the human hbr and uhr samples from the MAQC dataset. Table S2, Numbers of reads for the mouse neurosphere samples for treatment groups of K and N (the K_N dataset). Table S3, The number of reads for each individual sample of the LCL3 dataset. Table S4, The definition for TP, FP, TN, FN, TPR and FPR. Table S5, The false positive rate for Cuffdiff2, DESeq and edgeR based on the LCL1 dataset. (ZIP) [file pone.0103207.s001.zip › Supporting Information S1/Figure S3.tif]
